# Pathogenic copy number variants and *SCN1A* mutations in patients with intellectual disability and childhood-onset epilepsy

**DOI:** 10.1186/s12881-016-0294-2

**Published:** 2016-04-26

**Authors:** Andrew E. Fry, Elliott Rees, Rose Thompson, Kiran Mantripragada, Penny Blake, Glyn Jones, Sian Morgan, Sian Jose, Hood Mugalaasi, Hayley Archer, Emma McCann, Angus Clarke, Clare Taylor, Sally Davies, Frances Gibbon, Johann Te Water Naude, Louise Hartley, Gareth Thomas, Catharine White, Jaya Natarajan, Rhys H. Thomas, Cheney Drew, Seo-Kyung Chung, Mark I. Rees, Peter Holmans, Michael J. Owen, George Kirov, Daniela T. Pilz, Michael P. Kerr

**Affiliations:** Institute of Medial Genetics, University Hospital of Wales, Cardiff, CF14 4XW UK; Institute of Cancer and Genetics, Cardiff University, Cardiff, CF14 4XN UK; MRC Centre for Neuropsychiatric Genetics and Genomics, Institute of Psychological Medicine and Clinical Neurosciences, Cardiff University, Cardiff, CF24 4HQ UK; Llwyneryr Unit, Learning Disability Services, Clasemont Road, Morriston, Swansea, SA6 6AH UK; Learning Disabilities Directorate, Abertawe Bro Morgannwg University NHS Trust, Treseder Way, Caerau, Cardiff CF5 5WF UK; Department of Clinical Genetics, Glan Clwyd Hospital, Betsi Cadwaladr University Health Board, Rhyl, Denbighshire LL18 5UJ UK; Department of Paediatric Neurology, University Hospital of Wales, Cardiff, CF14 4XW UK; Department of Paediatric Neurology, Morriston Hospital, Abertawe Bro Morgannwg University Health Board, Swansea, SA6 6NL UK; Department of Paediatrics, Royal Glamorgan Hospital, Cwm Taf University Health Board, Pontyclun, Mid Glamorgan CF72 8XR UK; Welsh Epilepsy Centre, Neurosciences Directorate, University Hospital of Wales, Cardiff, CF14 4XW UK; Neurology and Molecular Neuroscience Research, Institute of Life Science, College of Medicine, Swansea University, Swansea, SA2 8PP UK

**Keywords:** Array comparative genomic hybridization, Intellectual disability, Epilepsy, Copy number variation, *SCN1A*

## Abstract

**Background:**

Copy number variants (CNVs) have been linked to neurodevelopmental disorders such as intellectual disability (ID), autism, epilepsy and psychiatric disease. There are few studies of CNVs in patients with both ID and epilepsy.

**Methods:**

We evaluated the range of rare CNVs found in 80 Welsh patients with ID or developmental delay (DD), and childhood-onset epilepsy. We performed molecular cytogenetic testing by single nucleotide polymorphism array or microarray-based comparative genome hybridisation.

**Results:**

8.8 % (7/80) of the patients had at least one rare CNVs that was considered to be pathogenic or likely pathogenic. The CNVs involved known disease genes (*EHMT1*, *MBD5* and *SCN1A*) and imbalances in genomic regions associated with neurodevelopmental disorders (16p11.2, 16p13.11 and 2q13). Prompted by the observation of two deletions disrupting *SCN1A* we undertook further testing of this gene in selected patients. This led to the identification of four pathogenic *SCN1A* mutations in our cohort.

**Conclusions:**

We identified five rare *de novo* deletions and confirmed the clinical utility of array analysis in patients with ID/DD and childhood-onset epilepsy. This report adds to our clinical understanding of these rare genomic disorders and highlights *SCN1A* mutations as a cause of ID and epilepsy, which can easily be overlooked in adults.

**Electronic supplementary material:**

The online version of this article (doi:10.1186/s12881-016-0294-2) contains supplementary material, which is available to authorized users.

## Background

Copy number variants (CNVs; chromosomal deletions and duplications) have been identified as significant aetiological factors in a range of neurodevelopmental disorders including intellectual disability (ID) [1], autism [2], epilepsy [3] and psychiatric disease [4]. The detection of a causative CNV in a patient is valuable for genetic counselling and, in some cases, guiding clinical management. The observation of a rare chromosomal abnormality in a patient with a rare neurological phenotype has occasionally been the vital clue leading to the identification of genes and pathways critical to brain development [5, 6]. A limited number of previous genome-wide CNV studies have focused on patients with both epilepsy and ID [7–10]. We set out to investigate the rare CNVs present in a series of 80 patients with ID/developmental delay (DD) and childhood-onset epilepsy. Our aims were: to determine the frequency of pathogenic CNVs in the cohort; to define the clinical features of patients carrying pathogenic CNVs; to identify any sub-groups of patients particularly enriched for pathogenic CNVs; and to highlight candidate genes for epilepsy and ID/DD.

## Methods

### Study subjects

Participants were recruited between 2010 and 2014. Participants were 80 unrelated patients (49 adults and 31 children) identified through medical genetics, learning disability and paediatric neurology clinics around Wales (see Additional file 1: Table S1 for further demographic information). Participants lacked a molecular diagnosis and had not previously undergone high resolution genome-wide cytogenetic analysis (<1 Mb resolution). The majority of participants had previously been tested by karyotype (61/80) combined with additional cytogenetic and molecular tests (Additional file 1: Table S2). Patients with known significant congenital brain malformations were excluded (e.g. malformations of cortical development, porencephaly, holoprosencephaly or intracerebral vascular malformations). CNV rates in the general population were estimated from 929 control subjects derived from the Wellcome Trust Case Control Consortium 2 National Blood Donors Cohort [11]. These were blood donors recruited by UK Blood Services and are therefore similar in ethnic origin to our mostly white British cohort. Controls were genotyped on Illumina OmniExpress single nucleotide polymorphism (SNP)-arrays.

### Ethics approval and consent to participate

The study was approved by the Research Ethics Committee for Wales (09/MRE09/51). Informed consent for testing and publication was obtained from all participants (or their parents/legal guardians).

### Microarray analysis

Genomic DNA was extracted from blood (*n =* 73) or saliva (*n =* 7). Samples were tested on one of three platforms: (i) Illumina610-Quad SNP-array (*n =* 20); (ii) Illumina OmniExpress SNP-array (*n =* 36); or (iii) microarray-based comparative genomic hybridization (array CGH) using a BlueGnome CytoChip ISCA 8x60k v2.0 array (*n =* 24). Validation testing was performed by fluorescent *in situ* hybridisation, multiplex ligation-dependent probe amplification (MLPA) or by testing on a second array platform. The method for identifying CNVs depended on the array platform. SNP-array data was called using PennCNV [12]. Called CNVs were filtered by probe number (10 or more) and gene content (at least one). We excluded CNVs which had 50 % or greater overlap with a CNV in the control cohort. However, for key genomic regions known to harbour recurrent CNVs associated with neurodevelopmental disorders which demonstrate incomplete penetrance (1q21.1, 15q11.2, 15q13.3, 16p11.2 and 16p13.11) we allowed CNVs to be present at low frequency in controls (<1 %). Analysis focused on deletions and duplications larger than 100 kb and 250 kb respectively (50 kb for disease regions). Array CGH data was referenced against same sex control DNA (Promega) and analysed using Illumina BlueFuse Multi (v3.1) software, with data filtered on consecutive probes (3 or more) and size (as above). Imbalances detected by array CGH were interpreted by comparison with data from the Database of Genomic Variants, International Standards for Cytogenomic Arrays consortium and local laboratory data. Coordinates are based on hg19/GRCh37. Statistical comparisons were made using Fisher’s exact test calculated with an online tool [13]. Parents and additional family members were analysed, where available, to determine if a CNV had arisen *de novo* or segregated with disease in a family. We assessed the clinical significance of CNVs based on their size, type, inheritance and whether they contained known disease genes. We were guided by the approach set out in previous publications [7, 14]. Based on this assessment some CNVs were annotated as ‘pathogenic’ (e.g. a *de novo* deletion of a proven disease gene/region) or ‘likely pathogenic’ (e.g. large CNVs containing genes/regions previously linked to disease). Other CNVs were considered to be of unknown significance.

### *SCN1A* gene testing

A subgroup of patients was tested for intragenic *SCN1A* mutations. Sequencing of the complete coding region and flanking sequence of the gene was performed by bidirectional Sanger sequencing (*n =* 4) or by targeted next-generation sequencing (NGS) (*n =* 11). Sequencing (Sanger or NGS) covered all the coding sequence of *SCN1A* along with 20 bp of flanking intron or untranslated region (UTRs). Sequencing did not cover the promoter, deep intronic regions or the rest of the UTRs. *In silico* analysis of detected variants included PhyloP [15], SIFT [16], Grantham distance [17], PolyPhen-2 [18] and CADD [19]. We also searched the Exome Aggregation Consortium (ExAC) database [20], dbSNP [21], ClinVar [22] and an *SCN1A* mutation-specific database [23]. Nucleotide and protein positions are based on NCBI Reference Sequences NM_001165963.1 and NP_001159435.1 respectively [24].

## Results and discussion

The 80 patients had a range of epilepsy phenotypes including epileptic encephalopathy (EE, *n =* 25), non-lesional focal epilepsies (*n =* 22), and genetic generalised epilepsy with ID (GGE-ID, *n =* 22) (Table 1). In the remainder, the epilepsy phenotype was unclassified or unknown. We found 22.5 % (18/80) of the cohort carried at least one rare CNV (Table 2). Three patients had more than one rare CNV. The average size of the CNVs was 647 kb (median 488 kb). We identified 8 CNVs considered to be likely (*n =* 3) or clearly pathogenic (*n =* 5) (Table 2). One patient (R660) had one clearly and one likely pathogenic CNV. This meant 7 (8.8 %) of our patients had pathogenic or likely pathogenic CNVs. Additional rare variants of uncertain clinical significance (VUS) were present in 11 further patients. We compared the frequency of CNVs in patients and controls. We found that large (>500 kb) low frequency (<1 %) genic CNVs were marginally more common in patients (13 %, 10/80) compared with controls (11 %, 105/929). However, this difference was not statistically significant (*P* = 0.71). The majority of patients had previously been tested by karyotype which will have depleted larger CNVs from the cohort.

**Table 1:** Epilepsy syndromes in the cohort at recruitment

Syndrome	Number
Epileptic encephalopathy (EE)	
Lennox-Gastaut syndrome	9
Dravet syndrome	3
West syndrome	2
Myoclonic astatic epilepsy	2
Epilepsy of infancy with migrating focal seizures	2
Ohtahara syndrome	1
Epilepsy with continuous spikes and waves during sleep	1
Unclassified EE with onset in infancy	5
Genetic generalised epilepsy with intellectual disability (GGE-ID)	
Myoclonic epilepsy	3
Progressive myoclonic epilepsy	1
Other GGE-IDs	18
Non-lesional focal epilepsies	22
Unclassified epilepsy	2
Unknown	9
Total	80

**Table 2:** Rare CNVs detected in 80 patients with ID/DD and epilepsy

Subject	Age	Sex	Clinical features	Seizure onset	Syndrome	Seizure types	Cytoband	CNV Type	Coordinates	Size (Kb)	Tests	Status	Interpretation	Genes
R125	10 m	F	Severe DD, cleft palate	3 m	EIMFS	FE, EBCS, CSE	2q24.3	Del	163823021–167958723	4,136	c/f	DN	Path	*SCN3A, SCN2A, SCN1A, SCN9A, SCN7A* & 8 others
R351	15y	M	Moderate DD, poor coordination, joint contractures, mildly dysmorphic	3 m	Dravet	FS, GTCS, CSE, M	2q24.3	Del	166842637–166918932	76	c/d	DN	Path	*SCN1A*
R404	7y	F	Mild DD, ASD	8 m	West	IS, Abs	16p11.2	Del	29595483–30198151	603	b/e,f	DN	Path	*DOC2A, KIF22, MAPK3, PRRT2, QPRT, SEZ6L2* & 24 others
R660	21y	M	Mod-severe ID, challenging behaviour, ASD, depression, dysmorphic	8 m	GGE-ID	Abs, M, FDS, EBCS	9q34.3	Del	140707889–140890373	182	b/e	DN	Path	*CACNA1B, EHMT1*
							3p14.2	Dup	59736299–61023355	1,287	b/e	Pat	Likely	*FHIT*
							3p22.1	Del	41359533–41824555	465	b/e	Mat	VUS	*ULK4*
R911	22y	F	Mod ID, small head, mildly dysmorphic	10y	FE	FDS, GTCS	2q22.3	Del	148691873–148818437	127	b/e	DN	Path	*MBD5, ORC4*
R913	20y	M	Mod-severe ID, challenging behaviour, ASD	10 m	FE	FS, FDS, EBCS	16p13.11	Dup	15512574–16262571	750	b/e	Mat	Likely	*ABCC1, C16orf45, FOPNL, KIAA0430, MIR484, MYH11, NDE1*
R345	27y	F	Mild ID, dysmorphic	<6y	GGE-ID	M, Abs, GTCS	2q13	Del	111392259–113094793	1,703	b/e	Pat	Likely	*BUB1, BCL2L11, ANAPC1, MERTK, FBLN7* & 5 others
R58	26y	F	Severe ID, scoliosis	<8y	GGE-ID	At, Abs, M	1q21.1	Dup	145625979–145723645	98	a/e	Mat	VUS	*CD160, RNF115*
R74^a^	51y	F	Mild-mod ID, depression	3 m	FE	FS, FE, EBCS	1p21.1	Del	104167778–104297867	130	a/e	U	VUS	*AMY1A, AMY1B, AMY1C, AMY2A*
R101	32y	M	ID, seizures	<16y	U	U	11q22.3	Del	109173027–109325299	152	b/e	Pat	VUS	*C11orf87*
R198	19y	M	Severe ID, ASD, mild right hemiparesis	7 m	LGS	FE, IS, Abs, NCS, GTCS, At, FDS	Xq28	Del	150589930–150811921	222	c/nd	U	VUS	*PASD1*
R528	23y	M	Severe ID, challenging behaviour, ASD, dysmorphic, regression	11y	FE	FE, Abs	15q13.3	Dup	32019919–32514341	494	b/e	U	VUS	*CHRNA7*
R605	41y	M	ID, seizures	<16y	U	U	15q11.2	Dup	22383292–23272733	889	b/e	Pat	VUS	*CYFIP1, NIPA1, NIPA2, TUBGCP5* & 8 others
							8p23.1	Del	11713852–12204679	491	b/e	Mat	VUS	*CTSB, FAM66D, FAM86B1, USP17L2, ZNF705D* & 6 others
R622^a^	28y	F	Moderate ID, challenging behaviour	6 m	GGE-ID	IS, GTCS, M	18p11.22	Dup	10042023–10581304	539	b/e	Mat	VUS	*APCDD1, NAPG*
R650	21y	M	Mild ID, thin habitus, depression	18 m	GGE-ID	Abs, M, GTCS	15q13.3	Dup	32029693–32514926	485	a/nd	U	VUS	*CHRNA7*
							15q14	Del	34700297–34807869	108	a/nd	U	VUS	*GOLGA8A*
R786	9y	M	Moderate DD, Leg hypertonia, dystonia	2y	GGE-ID (M)	M, Abs, At	21q21.3	Del	27715263–27955385	240	a/e	Mat	VUS	*CYYR1*
R931	15y	F	Severe DD, ASD, dysmorphic, microcephaly	12y	GGE-ID	GTCS	7q11.22	Del	71815170–72305671	491	b/e	Pat	VUS	*CALN1, MIR4650-1, MIR4650-2, SBDSP1, TYW1B*
R981	5y	F	Severe DD, regression, ASD, leg hypertonia	1w	GGE-ID	Abs, At, M, T	3p26.3	Dup	726675-1301830	575	c/nd	U	VUS	*CNTN6*

Age (at recruitment) and seizure onset in y(ears), m(onths) or w(eeks). Clinical features: *ID* intellectual disability, DD, developmental delay, *ASD* autism spectrum disorder

Syndrome, electroclinical syndrome or main epilepsy type at recruitment: Dravet, Dravet syndrome; EIMFS, epilepsy of infancy with migrating focal seizures: *FE* focal epilepsy, *GGE-ID*, genetic generalised epilepsy with ID, *LGS* Lennox-Gastaut syndrome, *U* unknown, *West* West syndrome

Seizure types: *Abs* absence, *At* atonic, *CSE* convulsive status epilepticus, *EBCS* evolution to bilateral or convulsive seizures, *FDS* focal dyscognitive seizures, *FS* febrile seizures, *GTCS* generalised tonic-clonic seizures, *IS* infantile spasms, *M* myoclonic, *NCS* non-convulsive status epilepticus, *T* tonic, seizure type at presentation is underlined (when known)

CNV type, Dup(lication) or Del(eletion). Coordinates, chromosome position of first/last abnormal probes based on hg19/GRCh37. Tests, primary array/confirmation method: (a) Illumina610-Quad SNP-array, (b) Illumina OmniExpress SNP-array, (c) BlueGnome CytoChip array CGH, (d) quantitative PCR, (e) Illumina Exome BeadChip or custom Illumina SNP array, (f) fluorescence *in situ* hybridization, and (nd) not done. Status: DN, *de novo*; inherited Pat(ernally); Mat(ernally) or U(nknown). Interpretation (of clinical significance): Path(ogenic); Likely, likely pathogenic; VUS, variant of uncertain significance

^a^Patients R622 and R74 had pathogenic *SCN1A* mutations which suggests these two CNVs are likely to be benign

### Pathogenic CNVs

The five clearly pathogenic CNVs were all *de novo* deletions. We found a *de novo* 127 kb deletion of 2q23.1 in a woman with moderate ID, mildly dysmorphic facial features (long face, thin upper lip, slightly upslanting palpebral fissures, long nose) and seizures. The deletion disrupted the first two non-coding exons of the *MBD5* gene. *MBD5* encodes a member of the methyl-CpG-binding domain family. The MBD5 protein binds to methylated DNA and is thought to regulate gene expression by controlling chromatin modification [25]. Deletions of the 5′- UTR of *MBD5* result in reduced expression of the gene [26]. Common clinical features in *MBD5* patients include ID/DD, seizures, language impairment, microcephaly, mild craniofacial dysmorphism and autism spectrum disorders (ASD) [26–28]. Interestingly, patients with CNVs confined to the 5′-UTR (like R911) have phenotypes similar to patients with larger 2q23.1 deletions. This highlights the critical impact of non-coding sequence at the locus [29].

We observed a *de novo* 182 kb deletion at 9q34.3 involving *EHMT1* in an adult male (R660) with moderate-to-severe ID, dysmorphic features (hypertelorism, mid face hypoplasia, prognathism), aggressive behaviour, autistic features, depression and epilepsy. Deletions at 9q34 involving *EHMT1* are responsible for Kleefstra syndrome [30]. *EHMT1* encodes a histone methyltransferase involved in transcriptional repression. EHMT1 is known to interact with MBD5 and they work together to regulate gene expression [25]. Characteristic features of Kleefstra syndrome include ID/DD, microcephaly, psychiatric disorders, severe behavioural problems, dysmorphic features, hypotonia, heart defects and seizures [31]. In addition to truncating *EHMT1* the 9q34 deletion involved the adjacent *CACNA1B* gene. *CACNA1B* encodes a subunit of a voltage-dependent calcium channel expressed on neurons. Mutations in other N-type voltage-dependent calcium channel subunits have been linked to a wide range of paroxysmal disorders including periodic paralysis [32], familial hemiplegic migraine [33], myoclonus-dystonia syndrome [34], childhood absence epilepsy [35] and idiopathic generalized epilepsy [36]. Therefore, it is possible that haploinsufficiency of *CACNA1B* may have contributed to the patient’s epilepsy phenotype. Patient R660 also had a paternally-inherited 1.3 Mb duplication involving the *FHIT* gene (considered to be likely pathogenic). The *FHIT* gene is a member of the histidine triad gene family. *FHIT* encodes diadenosine 5′,5‴-P1,P3-triphosphate hydrolase, an enzyme involved in purine metabolism. Rare CNVs involving *FHIT* have previously been described in autism [37, 38]. R660 carried a third rare CNV, a maternally-inherited 465 kb deletion at 3p22.1 involving *ULK4. ULK4* encodes a serine/threonine kinase. Expression of the *ULK4* gene is neuron-specific and developmentally regulated [39]. This third CNV was considered to be a VUS, although deletions in *ULK4* have recently been reported as a potential risk factor for schizophrenia [39].

The third clearly pathogenic CNV was a *de novo* 603 kb 16p11.2 deletion in a girl with mild DD, ASD and infantile spasms (seizure free following treatment). Seizures are a common feature of 16p11.2 deletion syndrome along with ASD, ID/DD, psychiatric disease and increased risk of obesity [40, 41]. The reciprocal duplications at 16p11.2 locus have also been associated with epilepsy including infantile spasms [7, 42]. The last two clearly pathogenic CNVs were both *de novo* deletions at 2q24.3: one was 76 kb in size and deleted exons 4 to 28 of the *SCN1A* gene; the other was 4.1 Mb and deleted 13 genes including *SCN1A. SCN1A* encodes a voltage-gated sodium channel which is essential for the generation and propagation of action potentials in neurons. Mutations in *SCN1A* cause a spectrum of seizure disorders including familial febrile seizures, generalised epilepsy with febrile seizures plus and Dravet syndrome (severe myoclonic seizures of infancy) [43–45]. Typical features of these disorders are seizure onset in infancy with fever sensitivity. Severe manifestations of *SCN1A*-related disease include pharmacoresistant seizures, ID/DD, ataxia and autistic behaviour [46, 47]. Patient R125, who had the larger of the two deletions, had a severe phenotype with poor seizure control, severe DD and a cleft palate. These additional features may be due to haploinsufficiency of other genes in the region. The deletion in R125 included *SCN2A*, *SCN3A* and *SCN9A*. All three of these genes encode voltage-gated sodium channels which have been linked to epilepsy [48–50]. The patient’s epilepsy phenotype was considered to be epilepsy of infancy with migrating focal seizures (EIMFS). A number of patients with 2q24.3 deletions and EIMFS -like phenotypes have recently been reported [51, 52]. Patient R351, who had the smaller of the 2q24.3 deletions, had previously undergone *SCN1A* sequencing which had not detected their multi-exon deletion. This highlights that DNA sequencing alone is insensitive to CNVs and that dose-sensitive techniques (e.g. array CGH or MLPA) are required to detect a significant proportion of *SCN1A* mutations [53].

Two further likely pathogenic CNVs were found. One was a paternally-inherited 1.7 Mb deletion of 2q13 in a female patient (R345) with mild ID, small ventricular septal defect, facial dysmorphism (long face, retrognathism, broad nasal root, hypertelorism, mild facial asymmetry) and epilepsy. Deletions at 2q13, similar to the one found in patient R345 have been reported in other patients with DD/ID [54, 55]. Common manifestations include facial dysmorphism, autistic features, seizures and cardiac malformations. Previously reported 2q13 deletions have been inherited from an apparently normal parent, consistent with incomplete penetrance. Interestingly, the father of R345 shares similar facial features, but has no history of ID or epilepsy. The third likely pathogenic CNV was a maternally-inherited 750 kb duplication of 16p13.11 in a man with mild ID, ASD, seizures and a history of aggressive episodes. We considered the 16p13.11 duplication to be likely contributory as there was a family history of childhood epilepsy in the patient’s mother and a maternal uncle (untested). Deletions in the 16p13.11 region are clear risk factors for neurodevelopmental disorders including epilepsy [3, 56]. There is also evidence that duplications at 16p13.11 predispose to neurodevelopmental disorders (ASD, schizophrenia and ID) [57–60] and have been reported in patients with epilepsy [61]. Several further CNVs at genomic ‘hot spots’ were observed (duplications at 1q21.1, 15q11.2 and 15q13.3). These duplications were all inherited from unaffected parents and overlapped CNVs in the control cohort. They were therefore considered to be VUS. It remains possible that some of these VUS have contributed to disease risk. For example, there is evidence that *CHRNA7* duplications may subtly increase the risk of neurodevelopmental disorders including ID [62]. However, further large-scale epidemiological studies are required to fully define these risks. Among the non-‘hotspot’ CNVs of uncertain significance we found a 575 kb duplication involving the first 4 exons of *CNTN6*. This duplication was identified in a 5-year-old girl with severe DD, ASD, bilateral lower limb hypertonia and early-onset seizures. *CNTN6* is an interesting candidate gene for neurodevelopmental disorders as it encodes a neural adhesion molecule that operates in the formation, maintenance and plasticity of neuronal networks. In addition, CNVs involving *CNTN6* have been reported in patients with DD/ID and autistic features [2, 63–65].

### *SCN1A* mutations

Struck by finding two deletions involving *SCN1A* we realised that this key monogenic cause of epilepsy had not been extensively pre-screened in our cohort (only 9/80). The majority of recruits were adults (*n =* 49) who were initially investigated before *SCN1A* testing was available. Furthermore, in contrast to paediatric settings, the significance of *SCN1A* mutations for adult patients is often neglected [66], usually because key elements of early history (e.g. age of onset, initial seizure types) are not available. We therefore selected a group of patients with early-onset epilepsy for *SCN1A* sequencing. Of the 38 patients with seizure onset before 12 months, 6 had previously had normal testing for *SCN1A* while 3 others had pathogenic CNVs. Fifteen of the remaining 29 patients were prioritized for testing based on clinical features (e.g. a history of myoclonic or febrile seizures). This found 4 pathogenic *SCN1A* mutations (Table 3). All four patients had seizure onset in early infancy (6 months or before) and ongoing seizures despite anticonvulsant therapy. Three of the mutations were missense mutations. The fourth was a 4 base duplication leading to a frameshift early in the gene. *In silico* analysis indicated the missense mutations were all deleterious changes affecting conserved residues (Table 3). One missense mutation segregated with epilepsy and ID phenotypes in the patient’s family (the proband’s two affected siblings and their mildly-affected mother) the others were all *de novo*. In combination with the array data these results indicate that at least 6/80 (7 %) of our cohort had *SCN1A*-related seizure disorders.

**Table 3:** *SCN1A* mutations in the cohort

Subject	R622	R74	R710	R769
Age	28y	51y	24y	3y
Sex	F	F	F	F
Clinical features	Moderate ID, challenging behaviour	Mild-mod ID, depression	Moderate ID, ataxia, stroke-like episodes	Mod-severe DD, poor coordination
Seizure onset	6 m	3 m	6 m	5d
Syndrome	GGE-ID	FE	PME	CSWS
Seizure types	IS, GTCS, M	FS, FE, EBCS	C-CSE, M, FDS, EBCS	T, GTCS, CSE, FE, At, Abs, M
Genomic coordinates	Chr2 g.166915177 _166915180dup	Chr2 g.166915162 G > A	Chr2 g.166913001 G > C	Chr2 g.166848780 C > T
cDNA	c.283_286dup	c.301C > T	c.393C > G	c.5005G > A
Protein	p.Gly96Glufs*24	p.Arg101Trp	p.Ser131Arg	p.Ala1669Thr
Inheritance	*De novo*	*De novo*	Segregates with phenotype	*De novo*
PhyloP	-	0.91 (highly conserved)	0.89 (highly conserved)	0.86 (highly conserved)
Grantham distance	-	101 (moderate)	110 (moderate)	58 (small)
SIFT	-	0 (deleterious)	0.02 (deleterious)	0 (deleterious)
PolyPhen-2 (HumVar)	-	0.982 (probably damaging)	0.368 (benign)	1 (probably damaging)
CADD (PHRED-scaled)	-	34 (top 0.1 %)	22.3 (top 1 %)	26.1 (top 1 %)
ExAC frequency	0	0	0	0
dbSNP	-	rs121917965	-	-

Age (at recruitment) and seizure onset in y(ears), m(onths) or d(ays). Clinical features: *ID* intellectual disability, *DD* developmental delay

Syndrome, electroclinical syndrome or main epilepsy type at recruitment: CSWS, epilepsy with continuous spikes and waves during sleep; *FE* focal epilepsy, *GGE-ID* genetic generalised epilepsy with ID, *PME* progressive myoclonic epilepsy

Seizure types: *Abs* absence, *At* atonic, *C* clonic, *CSE* convulsive status epilepticus, *EBCS* evolution to bilateral or convulsive seizures, *FDS* focal dyscognitive seizures, *FS* febrile seizures, *GTCS* generalised tonic-clonic seizures, *IS* infantile spasms, *M* myoclonic, *T* tonic, seizure type at presentation is underlined. Coordinates are based on hg19/GRCh37. Nucleotide and protein reference sequences were NM_001165963.1 and NP_001159435.1

## Conclusions

We have reported the range of rare CNVs found in a series of 80 Welsh patients with childhood-onset epilepsy and ID/DD. We identified clearly or likely pathogenic CNVs in 7 (8.8 %) of the patients including 5 rare *de novo* deletions. Our results highlight key genes for brain development including drawing attention to *SCN1A* mutations in adults with early-onset pharmacoresistant epilepsy and ID. Our results contribute additional phenotypic descriptions for these rare genomic disorders and support the use of molecular cytogenetic analysis in the genetic evaluation of patients with ID/DD and epilepsy.

## Additional file

Additional file 1: Table S1. A detailed demographic description of the cohort. **Table S2.** Previous cytogenetic and molecular testing in the cohort. (DOCX 17 kb)

## Abbreviations

Array CGH: microarray-based comparative genomic hybridization
ASD: autism spectrum disorder
CNV: copy number variant
DD: developmental delay
EIMFS: epilepsy of infancy with migrating focal seizures
EE: epileptic encephalopathy
ID: intellectual disability
GGE: genetic generalised epilepsy
MLPA: multiplex ligation-dependent probe amplification
NCBI: national center for biotechnology information
NGS: next-generation sequencing
SNP: single nucleotide polymorphism
VUS: variant of uncertain clinical significance
