# Supplementary material for: Pathogenic copy number variants and SCN1A mutations in patients with intellectual disability and childhood-onset epilepsy
Source: BMC Med Genet. 2016 Apr 26;17:34. doi: 10.1186/s12881-016-0294-2 (PMC4845474; doi:10.1186/s12881-016-0294-2)
Supplement: Additional file 1: Table S1. — A detailed demographic description of the cohort. Table S2. Previous cytogenetic and molecular testing in the cohort. (DOCX 17 kb) [file 12881_2016_294_MOESM1_ESM.docx]

Additional file 1:

**Table S1**

**A detailed demographic description of the cohort**

| Age at recruitment | | <1y | 2 |
| --- | --- | --- | --- |
|  | | 1-5y | 13 |
|  |  | 6-10y | 10 |
|  |  | 11-15y | 6 |
|  |  | 16-20y | 10 |
|  |  | 21-30y | 24 |
|  |  | 31-40y | 5 |
|  |  | 41-50y | 8 |
|  |  | 51-60y | 2 |
|  |  |  |  |
| Gender | <16 years | Male | 14 |
|  | | Female | 17 |
|  | 16 or over | Male | 22 |
|  | | Female | 27 |
|  |  |  |  |
| Age at seizure onset | | <1m | 10 |
|  | | 1-6m | 18 |
|  |  | 7-12m | 10 |
|  |  | 13m-5y | 20 |
|  |  | 6-10y | 6 |
|  |  | 11-15y | 3 |
|  |  | Uncertain | 13 |
|  |  |  |  |
| Ethnic origin | | White British | 78 |
|  | | South Asian | 1 |
|  |  | Mixed White/South Asian | 1 |
|  |  |  |  |
| Parental consanguinity | | No | 78 |
|  | | Yes | 2 |
|  |  |  |  |
| Probands with similarly affected first-degree relative(s) | | | |
|  | | 0 relatives | 73 |
|  |  | 1 relatives | 6* |
|  |  | 3 relatives | 1** |

Abbreviations: Age at recruitment and seizure onset is in y(ears) or m(onths).

* All siblings.

** A parent and two affected siblings.

**Table S2**

**Previous cytogenetic and molecular testing in the cohort**

| Disorder/gene/test | Methodology | Number (out of 80) |
| --- | --- | --- |
| Karyotype | Lymphocyte culture, G banding | 61 |
| *FMR1* | PCR of CGG repeat | 24 |
| *MECP2* | Sequencing +/- MLPA* | 17 |
| *CDKL5* | Sequencing +/- MLPA | 16 |
| *ARX* | PCR of polyalanine expansions +/- sequencing | 14 |
| Angelman syndrome | Methylation testing (PCR of bisulphite modified DNA in SNRPN region) | 13 |
| *SCN1A* | Sequencing +/- MLPA | 9 |
| 22q11.2 | FISH | 6 |
| Subtelomeric screen | FISH | 5 |
| *FOXG1* | Sequencing and MLPA | 5 |
| 17p11.2 | FISH | 4 |
| 1p36 | FISH | 3 |
| *STXBP1* | Sequencing | 3 |
| *TCF4* | Sequencing and MLPA | 3 |
| *POLG* | Pyrosequencing of common mutations | 3 |
| mtDNA studies | Fluorescent restriction digest PCR (common mutations), long range PCR (major mtDNA rearrangements) and real-time PCR (mtDNA depletion) | 3 |
| Prader Willi syndrome | Methylation testing (PCR of bisulphite modified DNA in SNRPN region) | 2 |
| *CSTB* | PCR of dodecamer expansion | 2 |
| *SLC9A6* | Sequencing | 2 |
| *TSC1/TSC2* | Sequencing | 1 |
| 22q13 | FISH | 1 |
| *DRPLA* | PCR of CAG repeat | 1 |
| *HTT* | PCR of CAG repeat | 1 |
| *NF1* | Sequencing | 1 |
| *PCDH19* | Sequencing | 1 |
| *SLC2A1* | Sequencing and MLPA | 1 |

Abbreviations: FISH, fluorescent *in situ* hybridisation; MLPA, multiplex ligation-dependent probe amplification; mtDNA, mitochondrial DNA; PCR, polymerase chain reaction.

*Historically some gene tests only used sequencing. More recent versions of these tests have included MLPA as well.
